# Expression of concern: Randomized comparison of superovulation with letrozole vs. clomiphene citrate in an IUI program for women with recently surgically treated minimal to mild endometriosis

**DOI:** 10.1111/aogs.14872

**Published:** 2024-05-01

Hatem Abu Hashim , Mohamed El Rakhawy , Ibrahim Abd Elaal (2012), Randomized comparison of superovulation with letrozole vs. clomiphene citrate in an IUI program for women with recently surgically treated minimal to mild endometriosis. Acta Obstetricia et Gynecologica Scandinavica, 91: 338–345. 10.1111/j.1600-0412.2011.01346.x 22181973

This Expression of Concern is for the above article, published online on December 19, 2011, in Wiley Online Library (wileyonlinelibrary.com), and has been published by agreement between the journal's Editor‐in‐Chief, Ganesh Acharya, the Nordic Federation of Societies of Obstetrics and Gynecology, and John Wiley and Sons Ltd. The Expression of Concern has been agreed upon following concerns raised by a third party and further investigation by Wiley's Integrity Assurance and Case Resolution team which revealed concerns with the statistical analysis and the data presented in the article. An institutional investigation at Mansoura University into the reliability of the published clinical trial was requested in 2021. The author has previously provided a letter from Mansoura University, dated March 17, 2012, which confirms the author did work and publish studies between 2009 and 2012 at the institute. However, we were unsuccessful in receiving a direct response from Mansoura University with regard to the current investigation despite our attempts. The journal has decided to issue an Expression of Concern to alert the readers as the authenticity of the data could not be confirmed.

